# Characteristics of older cyclists with self-perceived needs for improvement in cycling competence: SiFAr trial

**DOI:** 10.1007/s41999-023-00765-2

**Published:** 2023-03-28

**Authors:** Veronika Keppner, Cornel C. Sieber, Ellen Freiberger, Robert Kob, Sebastian Krumpoch, Hanna M. Siebentritt

**Affiliations:** 1 Institute for Biomedicine of Aging (IBA), Friedrich‐Alexander-Universität Erlangen‐Nürnberg (FAU), Kobergerstr. 60, 90408 Nuremberg, Bavaria Germany; 2 Department of Medicine, Kantonsspital Winterthur, Winterthur, Switzerland

**Keywords:** Older adults, e-bike, Cycling, Cycling safety, Aging

## Abstract

**Background:**

Older cyclists are in great risk of being injured or killed in a traffic-related accident and their needs should find more consideration in safety guidelines, urban planning, and future intervention programs.

**Objective:**

The aim for this cross-sectional analysis was to comprehensively explore characteristics of community-dwelling cyclists aged 65 years and older with a self-perceived need to increase cycle competence.

**Methods:**

118 older adults (mean age 73.3 ± 5.2 years, 61% women) performed a standardized cycle course representing specific cycling skills. Additionally, health and functional assessments were carried out and characteristics regarding demographic, health, falls, bicycle equipment/type and cycling biography/behavior were obtained.

**Results:**

The majority (67.8%) of this community-dwelling adults reported being unsafe when cycling and 41.3% had a bicycle fall in the past year. More than half of the participants showed at least one limitation in each of the measured cycling skills. Women significantly had more frequent limitations in four of the cycling skills measured (*p ≤ *0.001) compared to men. While no significant differences were found for falls, health and functional characteristics, women and men differed significantly in terms of bicycle type, equipment, and perceived safety (*p < *0.001).

**Conclusion:**

Limitations in cycling should be compensated by preventive bicycle training and a safe cycling infrastructure. Bicycle fit, the wearing of bicycle helmets and promotion of a sense of security while cycling can further reduce accident risk and must find recognition in safety guidelines. In addition, educational initiatives have to dismantle gender-related bicycle stereotypes.

## Key Summary Points

**Aim:**

To explore characteristics of community-dwelling cyclists aged 65 years and older with a self-perceived need to increase cycling competence.

**Findings:**

The majority (68%) of this collective reported being unsafe when cycling and 41% had a bicycle fall in the past year. More than half of the participants showed at least one limitation in each of the measured cycling skills required for safe cycling.

**Message:**

Knowledge about cycling behavior, bicycle type and cycling competence of older cyclists facilitates the planning of intervention programs and road safety campaigns to prevent mobility losses and accidents.

## Introduction

In an aging society that faces the consequences of climate change, cycling as a convenient communal mode of transportation is gaining importance. Riding the bicycle is affordable, environmentally friendly, and has positive effects on public health [1, 2]. Performed regularly, it reduces the risk of all-cause mortality and a multitude of diseases in middle-aged and older persons [3]. Additionally, bicycle usage facilitates the maintenance of social networks and autonomy, having a beneficial impact on mental health and quality of life particularly in old age [4–6]. The development of the e-bike^1^ as a comfortable alternative to conventional bicycles has further increased the popularity of cycling in recent years [7]. Accompanying this trend, bicycle-related risks increase, and especially older cyclists are in great danger of being injured or killed in an accident, disregarding the fact that minor accidents are traditionally underrepresented in official statistics [5, 8–11]. The higher vulnerability of older cyclists is caused by the age-related decline of physical and cognitive function, which requires them to adapt their cycling and traffic behavior to their mental and physical abilities [7, 12]. Furthermore, these changes lead to a higher proportion of older cyclists reporting uncertainties compared to younger ones [13], which is particularly true for women [14]. Further studies suggest that gender might be a relevant factor for differences in cycling-related characteristics such as perceived constraints [15], risk behavior [16] and bicycle use [17], but a comprehensive study in an older cohort is lacking.

In the context of analyzing bicycle safety issues in older cohorts, researchers have focused on external factors like traffic-related risks [18], crash causation [10], environmental barriers [4] or strategies to protect cyclists [19]. In contrast, little emphasis has been placed on the characterization of older cyclists, particularly with regards to self-perceived insecurities and needs for improvement while cycling. The identification and prototypical description of a potentially vulnerable collective in terms of bicycle safety and accident risk with ultimate mobility loss could be valuable considering safety guidelines, urban planning, and future intervention programs. Therefore, our objective was to comprehensively explore characteristics and challenges of community-dwelling cyclists aged 65 years and older with a self-reported need to increase cycle competence. To this aim, we investigated different internal domains like health and cognitive status, fall history, cycling biography, preferred bicycle type/setting and cycling competence taking possible gender differences into account.

## Methods

### Study design and participants

This cross-sectional analysis is based on the baseline data of the “Safer Cycling in Older Age” (SiFAr) project, a parallel group, randomized controlled intervention study with a duration of 3 years aiming to improve cycle competence by a structured training program on the bicycle (June 2020- August 2022). The results of the intervention program will be reported elsewhere, a detailed description of the study design, power calculation and procedure can be found in the published study protocol [20]. In short, 127 community-dwelling older adults (65 years and older) living in the area of Nuremberg-Fürth-Erlangen were included in the study by fulfilling at least one of the following inclusion criteria: (1) beginners with the e-bike or (2) feeling self-reported unsteadiness when cycling or (3) uptaking cycling after a longer break. Long-term cyclists without subjectively reported limitations in cycling and persons with diseases that contradict safe participation in the intervention were excluded.

### Ethics and study registration

The study protocol was approved by the ethics committee of the Friedrich-Alexander-Universität Erlangen-Nürnberg and was performed in accordance with the guidelines published in the Declaration of Helsinki. The study was registered at ClinicalTrials.gov (identifier: NCT04362514). Written informed consent was obtained from every participant prior to the start of the assessments at the beginning of the baseline visit.

### Data collection and measures

Baseline data collection took place between April and June in person in the study center (participants’ characteristics, functional and psychological assessments) and in the outdoor cycle course (performance in the cycle course). All test appointments were scheduled as morning sessions. Demographics, health characteristics, falls, cycling biography & behavior as well as bicycle type & equipment were assessed by standardized questionnaires. Body weight and height were measured to calculate BMI (kg/m^2^). EQ-5D visual analog scale (vas; 0-100) was used to assess the subjective health status [21]. The Falls Efficacy Scale (FES-I) short form was performed to measure concerns about falling with higher scores (7-28) indicating stronger concerns [22].

#### Health and functional assessments

Functional status was evaluated with the Short Physical Performance Battery (SPPB) testing three different domains of physical function: balance (side-by-side, semi-tandem, tandem stand), usual gait speed (4 m), and strength in lower extremities (5-repetition sit-to-stand). As suggested by Guralnik et al. [23], a sum score (0–12) was calculated with a higher overall sum score indicating better physical performance. Participants who fully completed the balance domain were additionally tested to see if they could hold a single-leg stand for at least 10 s [24]. Cognitive function was assessed with the “Montreal-Cognitive Assessment” [25] (MoCA, 0–30) with a score of 26 or higher indicating normal cognitive function. The MoCA [26] as well as the SPPB [27] show good to excellent reliability.

#### Cycling skills assessment

Cycling performance was tested in a standardized cycle course with 7 tasks in the order specified (slalom, slow cycling, dismounting into a hula hoop and getting on the bicycle on both sides, cycling through a narrow alley, turning to the off-side, precise braking). Detailed description of the cycling course can be found in the published study protocol [20]. To provide the participant with the test instructions, the tester and participant first walked through the course. After a test run, errors were documented by trained study personnel within one measurement run and cross-checked using video recordings.

The tasks of the cycle course represent specific cycling skills that were combined when possible. Therefore, the cycle course tasks slow cycling and cycling through a narrow alley were added to the cycling skill lane keeping. Dismounting and mounting on the bike were combined for each side. The cycle course task slalom represents the cycling skill riding curves. Taken together, six cycling skills were defined: riding curves, lane keeping, dismounting/mounting to the right and left side, turning to the left side and precise braking. Each cycling skill was dichotomized (error in the respective cycle course task yes/no) to reflect whether limitations are present or not.

### Statistical analysis

Statistical analysis was performed using SPSS Version 28 (IBM SPSS Statistics, Chigaco, IL, USA).

Participants’ characteristics are presented as median and interquartile range (IQR) for continuous variables as they were not normally distributed. Dichotomous and categorical variables are shown as absolute numbers and percentages. Depending on the scaling of the respective variable, Chi-square tests or non-parametric Mann–Whitney *U* tests were used to test for significant differences between women and men. To correct for multiple testing, Bonferroni-Holm-adjustment of p value was applied (*p ≤ *0.001). For significant differences, effect size measures are reported that indicate the strength of association (Cramér’s *V*, φ_c_ or Pearson’s correlation coefficient, r; 0 = no association to 1 = perfect association).

## Results

### Demographics, health characteristics and fall biography

Of 127 eligible individuals, nine SiFAr participants were excluded due to missing data in the cycle course. Compared to the participants of the complete-cases sample (*n = *118), the participants of the drop-out sample were significantly older (72.9 vs 77.9 years; *p = *0.46), with no gender differences.

Participants’ characteristics are presented in Table 1 for the total sample and separately for women and men. The age of the participants was 72.9 years, 61% of the sample were women. The main reason for participating in the underlying SiFAr-study was unsteadiness while cycling (67.8%). The health characteristics describe a relatively healthy collective of older adults. In line with this, the participants generally had a good functional status (SPPB score 11.5), which was confirmed by the result that the majority (67.8%) was able to perform the single-leg stand for longer than 10 s.

**Table 1:** Participants’ characteristics

	Overall (*N = *118)		Women (*N = *72)		Men (*N = *46)	
	*N*/*M*	%/IQR	*N*/*M*	%/IQR	*N*/*M*	%/IQR
Demographics/participation reasons						
Age [years]	72.9	8.2	72.2	9.7	74.0	7.6
Living alone	48	40.7%	38	52.8%	10	21.7%
Unsteadiness while cycling (1)	80	67.8%	49	68.1%	31	67.4%
Beginners with the e-bike (2)^a^	5	4.2%	1	1.4%	4	8.7%
Uptaking cycling after a longer break (3)	7	5.9%	2	2.8%	5	10.9%
1&2	8	6.8%	6	8.3%	2	4.3%
1&3	18	15.3%	14	19.4%	4	8.7%
Health characteristics						
BMI [kg/m^2^]	25.5	5.1	25.1	6.5	26.2	3.3
Diseases (number)	2.0	2.0	2.0	2.0	2.0	2.0
Medication (number)	3.0	4.0	2.0	4.0	3.5	4.0
SPPB [Sumscore 0–12]	11.5	1.0	12.0	1.0	11.0	1.0
Single-leg stand ≥ 10 s	80	67.8%	48	66.6%	32	69.6%
MoCA	27.0	3.0	27.0	4.0	26.0	3.0
EQ-5D-vas [score 0–100]	80.0	15.0	80.0	15.0	80.0	16.0
FES-I [score 7–28]	7.5	2.0	8.0	2.0	7.0	1.0
Falls						
Fall in the past year (yes)	43	36.4%	24	33.3%	19	41.3%
Fall injury in the past year	26	60.5%	16	66.7%	10	52.6%
Number of falls in the past years	1.0	1.0	1.0	0	1.0	1.0
Bicycle fall since age 60 (yes)	63	53.4%	37	51.4%	26	56.5%
Bicycle fall in the past year (yes)	26	41.3%	14	37.8%	12	47.2%
Number of bicycle falls since age 60	2.0	1.0	2.0	1.0	1.5	1.0

*N* number, *M* median, *IQR* interquartile range, *BMI* body mass index, *SPPB* short physical performance battery, *MoCA* montreal cognitive assessment, *EQ-5D-vas* EurQol-5 Dimension Visual Analog Scale; *FES-I* Falls Efficacy Scale International

^a^Within the past year

A fall with the bicycle since the age of 60 occurred in 53.4% of all participants, a bicycle fall during the last year was reported by 41.3%.

There were no important differences between women and men.

### Cycling characteristics and bicycle equipment

The bicycle was mainly used for distances up to 10 km, more than half of the participants cycled at least 3–4 times a week (see Table 2). Bicycles were used for different reasons, mainly for leisure activities. 29.7% of all participants cycled less often than in the past because they felt more insecure. There was a significant difference (Chi^2^ = 23.2; *φ*_c_ = 0.44, *p < *0.001) between women (45.3%) and men (4.3%) in reporting to cycle less often because of insecurity. More leisure time (57.6%) and physical activity (63.9%) were cited as reasons for increased bicycle use compared to the past.

**Table 2:** Cycling characteristics, bicycle equipment and cycling skills

	Overall (*N = *118)		Women (*N = *72)		Men (*N = *46)	
	*N*/*M*	%/SD	*N*/*M*	%/SD	*N*/*M*	%/SD
Cycling biography and behavior						
Cycling start (age in years)	9.7	± 6.8	10.7	± 8.3	8	± 2.3
Cycling break (yes)	71	60.2%	47	65.3%	24	52.2%
Bicycle use						
Never—2 × per month	19	16.1%	15	21.1%	4	8.7%
1–2 × per week	26	22%	14	19.4%	12	26.1%
3–4 × per week	38	32.2%	24	33.3%	14	30.4%
(Almost) daily	35	29.7%	19	26.1%	16	34.8%
Cycled kilometers per use						
0–5 km	38	32.2%	26	36.1%	12	26.1%
5–10 km	47	39.8%	28	38.9%	19	41.3%
10–20 km	23	19.5%	14	19.4%	9	19.6%
> 20 km	10	8.5%	4	5.6%	6	13%
Purpose of bicycle use						
Transportation	108	91.5%	67	93.1%	41	89.1%
Leisure time	110	93.2%	67	93.1%	43	93.5%
Health considerations/promotion	100	84.7%	61	84.7%	33	71.7%
I cycle less often because of…						
Health limitations	21	17.8%	16	22.2%	5	10.9%
Insecurity*	35	29.7%	33	45.8%	2	4.3%
I cycle more often because of…						
More leisure time	68	57.6%	33	45.8%	35	76.1%
No car	14	11.9%	11	15.3%	3	6.5%
Physical activity	75	63.6%	39	54.2%	36	78.3%
Bicycle helmet (yes)	109	92.4%	64	88.9%	45	97.8%
Use of bicycle helmet						
Never	2	1.8%	1	1.6%	1	2.2%
Rarely	27	24.8%	18	28.1%	9	20%
Frequently/often	26	23.9%	16	25%	10	22.2%
Always	54	49.5%	29	45.3%	25	54.3%
Bicycle type and equipment						
Bicylce type						
Unmotorised	62	52.5%	36	50%	26	56.5%
Motorized (E-bike)	54	45.8%	34	47.2%	20	43.5%
Tricycle	2	1.7%	2	2.8%	0	0
Frame type/geometry						
Low-step frame*	75	63.6%	56	77.8%	19	41.3%
Mid-step frame	21	17.8%	16	22.2%	5	10.9%
High top tube*	22	18.6%	0	0	22	47.8%
Coaster brake (yes)*	41	34.7%	34	47.2%	7	15.2%
Limitations^a^ in cycling skills						
Riding curves*	83	70.3%	65	90.3%	18	39.1%
Lane keeping	65	55.1%	42	58.3%	23	50%
Dismounting/mounting to the right side*	88	74.6%	63	87.5%	25	54.3%
Dismounting/mounting to the left side*	86	72.9%	60	83.3%	26	56.5%
Turning to the left side	61	51.7%	39	54.2%	22	47.8%
Precise braking*	33	28%	28	38.9%	5	10.9%

*N* number, *M* Mean, *SD* standard deviation

*p  ≤  0.001

^a^ ≥ 1 error in the respective cycle course task

More than half of the participants had a non-motorized bicycle. 63.6% selected a bicycle with a low-step frame, women (77.8%) were significantly more likely to use a low-step frame than men (41.3%; Chi^2^ = 42.3; *φ*_c_ = 0.60; *p < *0.001). In addition, they significantly cycled more often with coaster brakes than men (47.2% vs. 15.2%; Chi^2^ = 12.7; *φ*_c_ = 0.33; *p < *0.001). Although 92.4% of participants owned a helmet, more than a quarter reported never or rarely using it when cycling.

### Limitations in cycling skills

With the exception of *precise braking*, more than half of the participants showed at least one limitation in each of the measured cycling skills (see Table 2). Limitations were most frequently observed in *riding curves* (70.3%), *dismounting/mounting to the right* (74.6%) and *left side* (72.9%). Limitations were least frequent (28%) during *precise braking.* Women showed significantly more frequent limitations in *riding curves* (Chi^2^ = 35.2; *φ*_c_ = − 0.55; *p < *0.001), *dismounting/mounting to the left* (Chi^2^ = 10.2; *φ*_c_ = − 0.29; *p = *0.001) and *right side* (Chi^2^ = 16.3; φ_c_ = − 0.37; *p < *0.001) and *precise braking* (Chi^2^ = 10.9; φ_c_ = -0.30; *p = *0.001) than men.

**Fig. 1:**
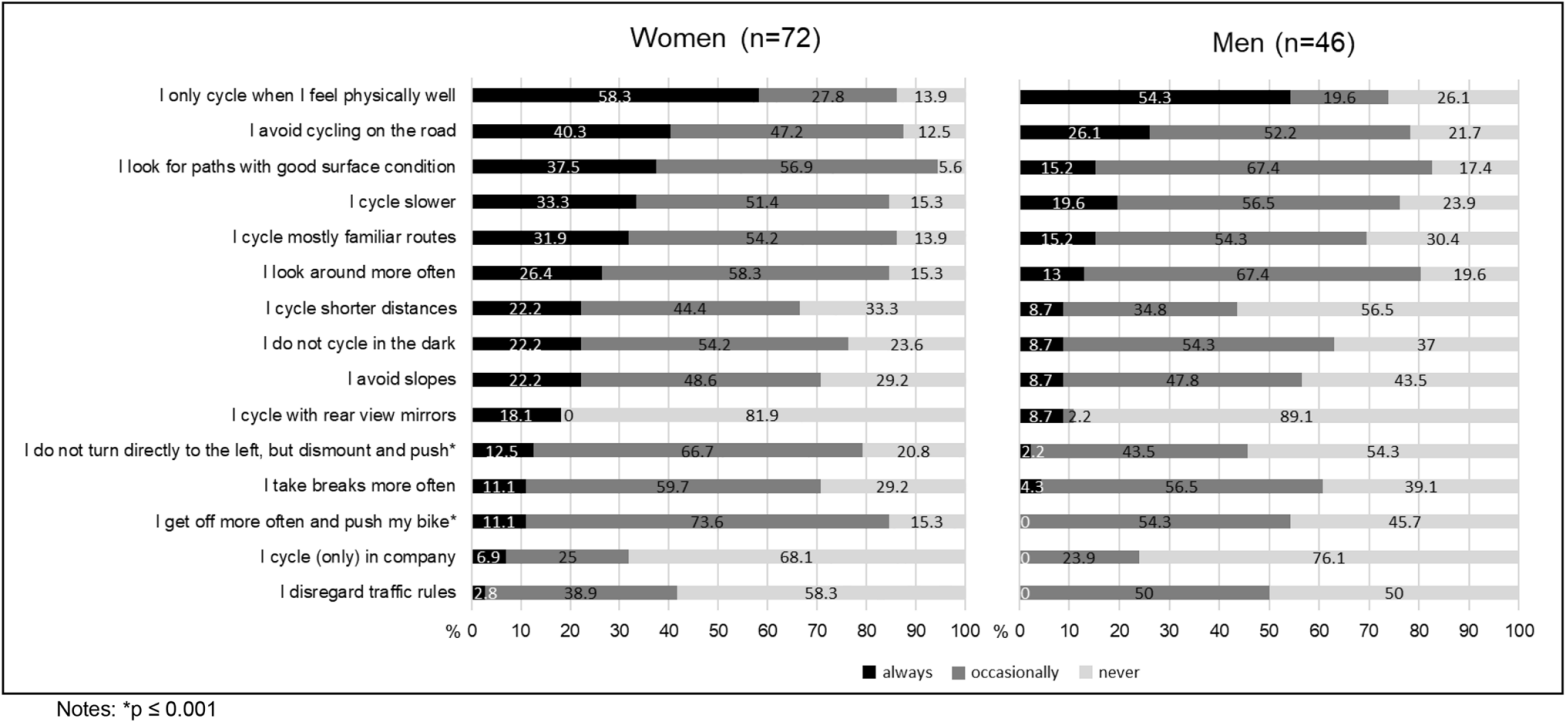
Use of compensatory cycling skills in women and men

As a sub analyses, we investigated via Chi-square tests whether the use of compensatory cycling strategies differed between women and men (see Fig. 1). The most reported strategy to compensate limitations while cycling is *to only cycle when I feel physically well*, which was true for both groups.

Descriptively, women seem to use all compensatory strategies more often men. However, if tested, significant differences were revealed for two strategies: Women reported significantly more often to *get off more often and push the bike* (Chi^2^ = 16.2; *φ*_c_ = 0.37; *p < *0.001) and to *not turn directly to the left, but dismount and push* (Chi^2^ = 15.5; *φ*_c_ = 0.36; *p < *0.001) compared to men.

## Discussion

The aim of this manuscript was a comprehensively description of community-dwelling older adults with self-perceived deficiencies in their cycling competence.

Our target group consisted of robust, independent, and overall healthy participants. This sub-study of the intervention trial SiFAr [20] showed a relatively high female proportion, which is consistent with findings of Sieverding [28] that women generally tend to be more interested in health promotion than their male counterparts. Although no important gender differences regarding cycling biography, behavior, and cycling-related falls were registered, women seem to perceive more environmental constraints when riding the bicycle [15]. In line with this, our female collective reported significantly more often to cycle less because of insecurities compared to male participants.

Nearly half of our study group had an e-bike, reflecting its increasing popularity, particularly among older adults [4, 7]. The descriptive and cross-sectional data showed no associations between e-bike use and limitations in cycle competence, which may be due to the fact that some cycle course tasks are easier to perform with an e-bike compared with an unmotorized bicycle and vice versa [20]. Women's bicycles were significantly more often equipped with coaster brakes, which could be attributed to the gender difference in grip strength [29]. Further gender-specific effects in terms of frame type might be a relic of historical bicycle design, as in the past, males’ heavier body weight had to be compensated by an additional high top tube. Also, men may prefer the sportier look compared to the low-step frame of the so-called “ladies bikes”. Even though our participants reported self-perceived deficiencies in their cycling competence, not all of them owned a bicycle helmet, and only two-thirds reported using it on a regular basis while cycling. As according to Zwipp et al. [18] and a road safety report of the Eurpoean Commission [11] especially older cyclists seem to reduce their risk of serious head injuries by wearing a helmet, its use should be emphasized more in preventive bicycle programs.

Albeit participants generally demonstrated a good functional status in our assessments, almost everyone of them showed limitations in the standardized cycle course. Since the cycle course simulates critical traffic situations associated with single-bicycle accident causes [7, 30], our results highlight an otherwise hard to identify at-risk population. In further reference to the limitations, several significant gender differences were observed. Compared to female participants, men had less problems mounting/dismounting their bicycles. This is in line with previous findings that women had significantly more problems, at least with the dismounting procedure [31]. The gender-related effects in braking precision could be explained with the usage of different brake systems. Women bicycles were significantly more often equipped with coaster brakes. It seems plausible that deceleration by hand brakes offers more control over modulation or braking power. More bicycle control could also be a reason why women showed more difficulties in *riding curves*. Additionally, the slalom task (i.e. *riding curves*) required challenging obstacle navigation and given the increased accident risk, female participants may have been more inclined to choose safer, but more penalizing cycling strategies. Several studies have proven that men tend to take more risks in everyday situations (i.e. health, recreation) [16]. Accordingly, significant gender effects were detected only in cycle course tasks that required higher risk taking.

The regular use of the majority of compensatory cycling skills in the context of everyday bicycle traffic was reported, indicating an overall need for improvement in cycle competence. Significant differences between women and men were present in the strategies of pushing the bicycle for compensation. However, the identified differences in using compensation strategies may indicate that women adapt to their lower competence level and their self-perceived unsteadiness when cycling. Interventions and health campaigns to promote cycling mobility should therefore also show alternatives and strategies to cope with major insecurities or limitations.

Although the current study provides additional insight regarding characteristics and challenges of older cyclists, it does have some limitations. For example, all data on characteristics and behavior are cross-sectionally, which is why no statements about their stability regarding a certain time frame are possible. No causal relation between reported need for improvement in cycle competence and objective limitations can be derived. Furthermore, due to the Bonferroni-Holm adjustment of the *p* value to correct for multiple testing, the sample size might be too small for sufficient statistical power (accumulation of type II error).

To date, no study has characterized cycling behavior, bicycle type and cycling performance of a potential at-risk group. Additional knowledge about bicycle characterization and cycling behavior should facilitate the planning of traffic safety campaigns and intervention programs. Since almost every participant showed limitations in our standardized cycle course and reported the use of compensatory cycling skills in everyday traffic, more emphasize should be placed on preventive bicycle training and safe cycling infrastructure. To further reduce accident risk, safety guidelines should particularly highlight bicycle fit, the wearing of bicycle helmets and promote a sense of security while cycling. In addition, gender-related bicycle stereotypes should be dismantled through educational initiatives.

## Data Availability

The datasets used and/or analysed during the study are available from the corresponding author on reasonable request.

## Footnotes

1 For this paper the term e-bike will be used exclusively to refer to electrically assisted bicycles which require the rider to pedal and with pedal assistance up to 25 km/h.
